# *In vitro* cytokine profiles and viability of different human cells treated with whole cell lysate of *Mycobacterium avium* subsp*. paratuberculosis*

**DOI:** 10.1186/1757-4749-4-10

**Published:** 2012-09-24

**Authors:** Pittu Sandhya Rani, Nikhil Kumar Tulsian, Leonardo A Sechi, Niyaz Ahmed

**Affiliations:** 1 Pathogen Biology Laboratory, Department of Biotechnology, School of Life Sciences, University of Hyderabad, Hyderabad, India; 2 Department of Biomedical Sciences, University of Sassari, Sassari, Italy; 3 Institute of Biological Sciences, University of Malaya, Kuala Lumpur, Malaysia

## Abstract

*Mycobacterium avium* subsp. *paratuberculosis* (MAP) is a zoonotic pathogen, a very slow growing bacterium which is difficult to isolate and passage in conventional laboratory culture. Although its association with Johne’s disease or paratuberculosis of cattle is well established, it has been only putatively linked to Crohn’s disease in humans. Further, MAP has been recently suggested to be a trigger for other autoimmune diseases such as type-1 diabetes mellitus (T1DM). Recently, some studies have indicated that exposure to MAP is associated with elevated levels of antibodies against MAP lysate although the exact mechanism and significance of the same remains unclear. Further, the cytokine profiles relevant in MAP associated diseases of humans and their exact role in the pathophysiology are not clearly known. We performed *in vitro* cytokine analyses after exposing different cultured human cells to the whole cell lysate of MAP and found that MAP lysate induces secretion of cytokines IL-1β, IL-6, IL-8, IL-10 and TNF-α by human peripheral blood mononuclear cells (PBMCs). Also, it induces secretion of IL-8 by cultured human stomach adenocarcinoma cells (AGS) and PANC-1(human pancreatic carcinoma cell line) cells. We also found that MAP lysate induced cytotoxicity in PANC-1cells. Collectively, these results provide a much needed base-line data set of cytokines broadly signifying a MAP induced cellular response by human cells.

## Introduction

*Mycobacterium avium* subsp. *paratuberculosis* (MAP) causes chronic inflammation of intestines in cattle and other ruminants, known as Johne’s disease (JD) [1]. MAP infection in JD leads to an early proinflammatory and cytotoxic response (Th-1 like) that further escalates to a predominant antibody based immune response (Th-2 like). Furthermore, studies have demonstrated that MAP infected cattle show enhanced IL-8 gene expression in intestinal tissues when compared to controls [2]. Studies on ovine macrophages indicated that MAP can induce low level apoptosis but not cytotoxicity (necrosis) [3]. MAP, an obligate zoonotic pathogen is also linked to a similar type of enteritis as JD, in humans, known as Crohn’s disease [4,5], wherein, the symptoms include abdominal pain, ulcers and diarrhoea (with bloody episodes) [6]. Moreover, this disease is also considered as an autoimmune disorder with inflammation contributed by Th1 cytokine responses [7]. Studies have also suggested that Crohn’s disease might have a genetic link such as mutation in the NOD2 gene [8]. The epidemiological studies have indicated that Crohn’s disease is more common in the temperate regions of the globe with intensive farming [9]. Diseased animals with clinical JD may shed MAP in their milk and faeces, and it is observed that MAP resists chlorination and pasteurization [10]. The existence of MAP in sphaeroplast form in addition to its bacillary form ensures survival [11]. ELISA studies have indicated correlation between MAP infection and type 1 diabetes mellitus (T1DM) [12,13]. It has been reported that children exposed to MAP in early life have low incidence of certain autoimmune diseases and exposure to MAP is associated with raised antibody levels against MAP [14-16].

In both cattle and humans, there is a long delay between the MAP infection and occurrence of the clinical disease. The clinical manifestation could ultimately be the outcome of various cytokine responses of an activated immune system unable to effectively control the infection [17]. Both Crohn’s disease and Type 1 diabetes are characterized by inflammatory responses, and, genetic predisposition alone cannot explain their acquisition. Several factors such as gut microflora, pathogens, dietary elements and environmental toxins are being considered in the development of these diseases although MAP has emerged as an important microbial trigger.

Given some association studies that putatively link MAP to T1DM or Crohn’s, not many efforts have been directed at identifying the cytokine profiles pathognomonic of MAP infection in humans. In this preliminary study we were interested to analyze cytokine coordinates relevant to chronic autoimmune disease pathology by *in vitro* studies of different human cells after treatment with whole cell lysate of MAP.

## Results and discussion

### Responses of the PBMCs

**Figure 1:**
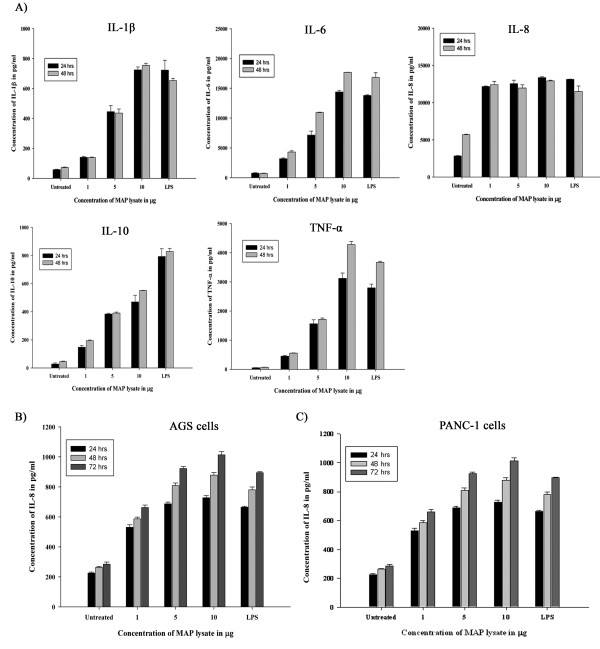
**(A) Cytokine analysis of culture supernatants of human peripheral blood mononuclear cells (PBMCs) upon treatment with MAP lysate indicated increased secretion of IL-1β, IL-6, IL-8, IL-10 and TNF-α (B) Cytokine analysis of culture supernatants of AGS cells resulted in increased secretion of IL-8 (C) Cytokine analysis of the culture supernatants of PANC-1 cells resulted in increased secretion of IL-8 in a dose and time dependent manner.** The statistical analysis (of untreated fractions and those with the treatment of different concentrations of MAP lysate and LPS) corresponding to PBMCs at 24 and 48 hr and AGS and PANC-1 at 24 hr, 48 hr and 72 hr resulted in p < 0.05 indicating significant increase in the secretion of cytokines.

We performed a set of experiments including cytokine analysis with MAP lysates. These included the treatment of human PBMCs with MAP lysate that resulted in an increased secretion of cytokines IL-1β, IL-6, IL-8, IL-10 and TNF-α in a dose and time dependent manner (Figure 1A). The cytokine responses from the human PBMCs were from two normal humans who presumably have not been exposed to MAP. Hence the cytokine responses were the innate ones. We do believe that these PBMCs may not be naïve to the mycobacterial antigens either because BCG vaccination is a normal practice in India or due to a saprophytic mycobacterial exposure. This could be one of the possible reasons as to why the PBMCs revealed highly significant cytokine responses.

### Responses of the cell lines

Treatment of AGS cells and PANC-1 cells with MAP lysate resulted in increased secretion of IL-8 in a dose and time dependent manner (Figure 1B and Figure 1C), but, secretion of other cytokines was not observed (data not shown). IL-8 is a chemotactic factor that mediates recruitment and activation of the cells of inflammation in the lesions of various types of inflammatory processes [18]. TNF-α and IL-1β are involved in Th-1 response; IL-10 is involved with Th-2 response. Although, IL-6 mediates acute phase response but when its function persists as proinflammatory cytokine, then acute inflammation turns into a chronic one. Further, in autoimmune disorders, IL-6 is not only involved in the maintenance of inflammation but also in modification of the immune responses [19]. These points suggest that MAP lysate activates both Th-1 and Th-2 responses. TNF-α secreted by macrophages induces inflammation; increased levels of TNF-α have been found to be associated with the development of Crohn’s disease [20]. Further, significantly higher concentrations of TNF-α were found in MAP-positive Crohn’s disease patients [21]. Plasma concentration of IL-6 was significantly high in patients with Crohn’s disease when compared to healthy controls [22]. Previous studies have shown that monocytes isolated directly from the blood of T1DM individuals secreted proinflammatory cytokines such as IL-1β and IL-6 thereby triggering the expansion of Th17 cells that are intimately involved in the development of autoimmune diseases [23]. Nevertheless, in patients with recent onset of T1DM and also in animal models of T1DM, increased expression of TNF-α and IL-1β was observed in pancreas [24]. We do not believe that the observed cytokine responses could be due to other confounding triggers (such as Mycobactin J used in bacterial culture media or the foetal bovine serum used in cell culture), as we have taken enough care to ensure fool proof protocols and ensured essential centrifugation and washing steps to move away any residual media or living bacterial cells.

### Cytotoxic effects of the MAP lysate

**Figure 2:**
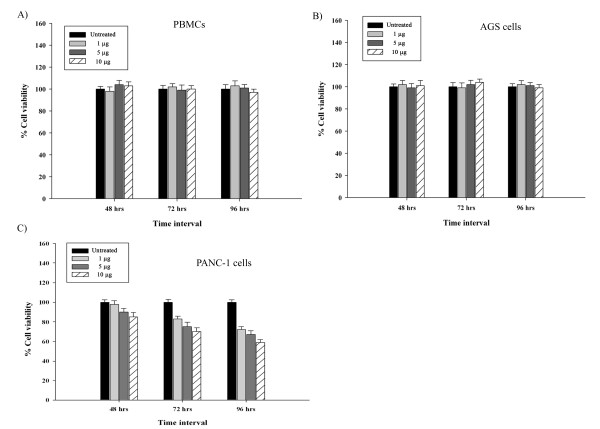
**(A) MTT assay of PBMCs indicating no cytotoxic effect upon treatment with MAP lysate (B) MTT assay of AGS cells after treatment with MAP lysate resulted in no cytotoxicity (C) MTT assay of PANC-1 cells on treatment with MAP lysate indicated no cytotoxicity at 48 hr but significant cytotoxicity was observed in PANC-1 cells at 72 hr and 96 hr.** The statistical analysis between untreated and with different concentration of MAP lysate of PBMCs and AGS cells at 48 hr, 72 hr and 96 hr resulted in p > 0.05 indicating there is no statistically significant cytotoxicity upon treatment with MAP lysate when compared to untreated. The statistical analysis between untreated fractions of PANC-1 and those treated with different concentrations of MAP lysate at 48 hr resulted in p > 0.05 indicating that there is no significant cytotoxicity on treatment with MAP lysate but the statistical analysis of PANC-1 untreated and treated with different concentration of MAP lysate at 72 hr and 96 hr indicated p < 0.05 indicating significant cytotoxicity. The OD_570_ values are represented as % cell viability and one of the OD_570_ of the untreated cells is taken as 100% viability for each cell types in MTT assay.

**Figure 3:**
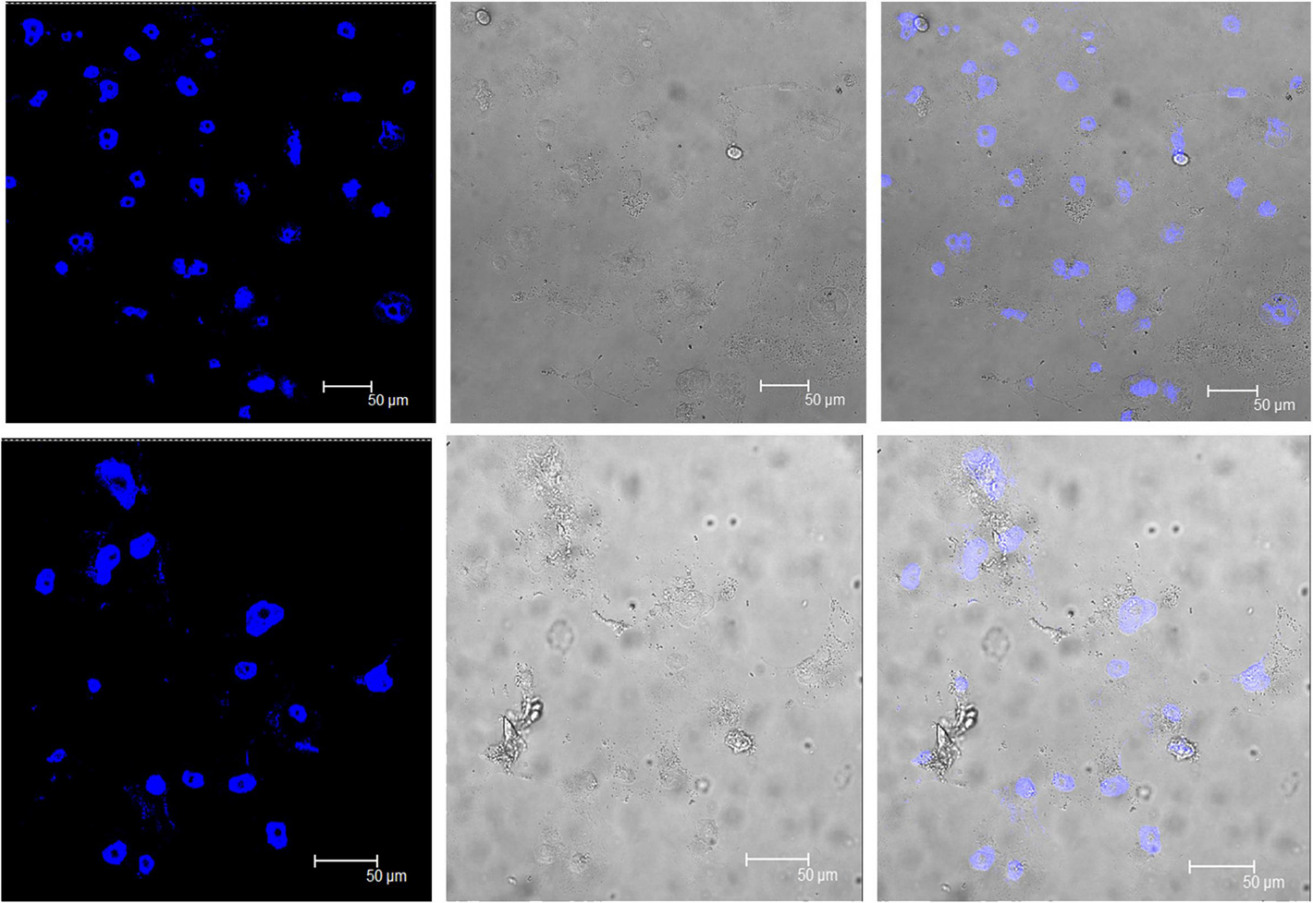
**DAPI stained PANC-1 cells on treatment with MAP lysate indicated cell membrane rupture representing necrosis when compared to untreated PANC-1 cells**.

To determine the possible cytotoxic effects of MAP lysate, we performed MTT assay with human PBMCs, AGS cells and PANC-1 cells after treatment with MAP lysate. The results indicated that MAP lysate caused no cytotoxic effects to the human PBMCs and AGS cells (Figure 2A and Figure 2B). However, when PANC-1 cells were treated with MAP lysate, no cytotoxic effect was observed up to 48 hr, but, a dose dependent cytotoxic effect was observed at 72 and 96 hr (Figure 2C). This was further confirmed by DAPI staining of PANC-1 cells on treatment with MAP lysate; this observation indicates cell membrane rupture representing necrosis (Figure 3). In view of these findings, we believe that MAP lysates exert differential effect on different types of epithelial cells i.e. AGS and PANC-1 cells. Further, cell viability was not affected in all the cell types when MTT assay was performed after treatment with other mycobacterial cell lysates such as the crude antigen preparation of *Mycobacterium smegmatis* (data not shown).

## Conclusions and prospective

Taken together, these observations portray cytokine coordinates of the treatment of cultured human cells with MAP lysate which were documented for diverse cell types using cytometry and imaging experiments. MAP lysate induced dose and time dependent cytotoxicity in PANC-1 cells. Further, we found whole cell lysate preparation to be an important ‘global’ antigen to perform cytokine assays probably indicative of the cellular responses of mammalian cells rather than employing individual MAP proteins to interact with cultured cells. The latter is both reductive and a time consuming proposal. Having this said, some issues remain. It is difficult to ascertain if the responses of MAP lysate-treated cells represent what occurs *in vivo* in MAP-associated diseases. Are these observations unique to MAP lysates? What are the bioactive components responsible for the observed response? Is the same response induced when the gut is challenged with MAP or the crude MAP preparations used for immunization? Although we do not have full answers to these obvious questions, it will be possible to continue work on these lines to come up with a more robust *in vitro* model of MAP infection in the near future.

## Materials and methods

### Ethics statement

This study did not entail any specific ethical approvals although informed consents were obtained from the 2 healthy donors of the human peripheral blood mononuclear cells (PBMCs).

### Preparation of MAP lysate

The MAP strain 1515(ATCC43015) was cultured in 7 H9 medium (Sigma-Aldrich) supplemented with 10% ADC (albumin dextrose catalase), 0.05% Tween80 (Sigma-Aldrich). and Mycobactin J (2 μg/ml) and incubated at 37°C until an approximate OD of 500 was achieved. The culture was then centrifuged at 4000 g for 20 min and the cell pellet washed twice with 1XPBS. The cells were subjected to disruption on ice by using ultrasonic homogenizer. The resultant lysate was then centrifuged at 10000 × g for 20 min to remove unbroken cells and cell debris. The supernatant was decanted and transferred into a fresh tube. The protein concentration was determined by Bradford’s reagent.

### Cytokine analysis of human PBMCs, AGS and PANC-1 cells

The heparinised blood was collected from two healthy individuals and PBMCs were isolated from the method previously described [25]. A total of about 0.5 million cells per ml of RPMI1640 media (Hyclone) containing 10% foetal bovine serum (FBS) (Hyclone) were treated with different concentrations of MAP lysate (1 μg, 5 μg, 10 μg) as compared to treatment by 1 μg of *E. coli* LPS (Sigma) used as a positive control. The culture supernatants were collected at 24 and 48 hr respectively and were stored at −80°C until the cytokine analysis was performed. A total of 0.5 million AGS cells (NCCS, Pune, India) per ml of HAMS F-12 media (Hyclone) containing 10% FBS and 0.5 million of PANC-1 cells (ATCC) per ml of DMEM high glucose (Hyclone) media containing 10% FBS were treated with different concentrations of MAP lysate (1 μg, 5 μg, 10 μg) and with 1 μg of LPS (positive control). The culture supernatants were collected at 24, 48 and 72 hr in each case and stored at −80°C until the cytokine analysis was performed. The cytokines IL-1β, IL-6, IL-8, IL-10, and TNF-α in the culture supernatants were analysed by BD CBA flex kit (BD Biosciences, USA). The acquisition of the sample was carried out in a BD FACS Canto II flow cytometer by using BD FACS diva software and the analysis was carried out by plotting the standard curve for each cytokine by using FCAP array software (Soft Flow/BD Biosciences).

### MTT (3-(4, 5-Dimethylthiazol-2-yl)-2, 5-diphenyl tetrazolium bromide) assay of PBMCs, AGS and PANC-1 cells upon treatment with MAP lysate

A total of about 0.05 million cells of human PBMCs per 200 μl of RPMI1640 media (Hyclone) containing 10% FBS (Hyclone), 0.05 million AGS cells per 200 μl of HAMS F-12 media (Hyclone) containing 10% FBS (Hyclone) and 0.05 million PANC-1 cells per 200 μl of DMEM High glucose (Hyclone) containing 10% FBS (Hyclone) were treated with different concentrations (1 μg, 5 μg, 10 μg) of MAP lysate. MTT assay was carried out after 48, 72 and 96 hr respectively. To the cells containing media, 50 μl of 5 mg/ml of MTT (Sigma) was added and incubated at 37°C for 4 hr.Then the plate was centrifuged at 2000 rpm for 10 min.The supernatant was discarded and 200 μl of 40 mM acidified isopropanol was added and incubated at 37°C for 15 min. The purple color thus formed was measured at 570 nm in ELISA reader (infinite M200 TECAN).

### DAPI (4′, 6-diamidino-2-phenylindole) staining of PANC-1 cells

A total of about 0.05 million of PANC-1 cells were grown on the cover slip and treated with 10 μg MAP lysate for 72 hr. The media from untreated cells and treated cells was removed and the cells washed with 1X PBS. The cells were then fixed by treatment with 4% paraformaldehyde and incubated for 15 min at 37°C. The cells were then washed thrice with 1X PBS. The cells were subsequently treated for 15 min at 37°C with 0.2% Triton-X 100 in PBS to induce permeability. The cells were then washed thrice with 1X PBS. DAPI with antifade reagent was subsequently added to the cells on the coverslip. The coverslip was then carefully reverted on to the clean glass slide and placed for observation under a fluorescence microscope (Leica).

### Statistical analysis

The student’s *t* –test was performed using Graph pad Prism 5.0 software. The *p-*value was calculated for each experiment which was conducted in triplicates.

## Competing interests

The authors declare that they have no competing interests.

## Authors’ contributions

PSR and NKT performed the experiments, analyzed results and wrote the draft manuscript. NA provided laboratory facilities and resources for the study and contributed to the discussions. LAS generated MAP lysates and provided suggestions for the interpretation of the results. All authors read and approved the final manuscript.
